# Superhydrophobic hBN-Regulated Sponges with Excellent Absorbency Fabricated Using a Green and Facile Method

**DOI:** 10.1038/srep45065

**Published:** 2017-03-23

**Authors:** Ying Zhou, Yao Wang, Tengfei Liu, Gang Xu, Guangming Chen, Huayi Li, Lichun Liu, Qiqi Zhuo, Jiaoxia Zhang, Chao Yan

**Affiliations:** 1 School of Materials Science and Engineering, Jiangsu University of Science and Technology, Zhenjiang, 212003, P R China; 2 School of Naval Architecture and Ocean Engineering, Jiangsu University of Science and Technology, Zhenjiang, 212003, P R China; 3 Institute of Chemistry, Chinese Academy of Science, Beijing, 100190, P R China; 4 College of Biological, Chemical Sciences and Engineering, Jiaxing University, Jiaxing, 314001, P R China

## Abstract

The world faces severe environmental, human and ecological problems when major oil spills and organic discharges are released into the environment. And so it is imperative to develop tools and high performance innovative materials that can efficiently absorb these organic discharges. Furthermore, green, facile methods to produce these advanced materials are also needed. In this paper, we demonstrate a novel porous supersponge based on melamine coated with hBN. This superhydrophobic sponge (with a contact angle >150°) exhibits excellent absorption performance for oils and organic solvents, including good selectivity, high capacity (up to 175 g·g^−1^) and extraordinary recyclability (less than 20% decline after 30 cycles of absorption/squeezing). The synthetic procedure required only ultrasonication and immersion of the sponge in aqueous hBN solution, being a green, cost-effective and scalable production methodology. By virtue of the straightforward and cost-effective fabrication method, along with the excellent absorption performance, hBN-decorated sponges have great promise for real world practical application in the field of oil spills and organic leakage cleanup.

With the development of industry, oil spills and other releases of organic pollution have become one of the most serious environmental and ecological problems. After an oil leak, the sea water is polluted by various organic molecules; these include alkanes, cycloparaffins and aromatic hydrocarbons all contained in crude oil, this happens in a short time^1^,^2^. Removal of an oil spillage and chemical leakage from water has become increasingly important according to the awareness of environmental protection and the impact long term contamination can have. The conventional methods such as oil boom or oil skimmer, physical diffusion (aided by dispersants), combustion, bioremediation technique, chemical reagent method^3^,^4^,^5^,^6^ have been used for oil removal to realize the water recovery, but they either show poor efficiency, low absorption capacity and poor recyclability or may introduce other types of containments during the clean-up procedure. Absorbent treatment^7^,^8^,^9^ is considered to be the most desirable option for the effective and environmentally friendly clean-up of oil pollution and water purification.

Recently, a variety of highly microporous materials that act as oil absorbers have attracted considerable interest in the field of clean-up of oil from water, as these can achieve oil−water separation via a simple, fast, and effective absorption process. Carbon aerogels^10^, CNT sponges^11^, graphene foams^12^,^13^, CNT and graphene-coated polymer sponges^14^,^15^ have been used to absorb oils and organic solvents from water. Benefitting from the high porosity of a 3D network and being hydrophobic, they display outstanding absorption performance. For example, the adsorption capacity of graphene foam for toluene and chloroform is up to 279 g.g^−1^ and 400 g.g^−1^, respectively, which is much higher than other conventional adsorbents^16^. In particular melamine sponges have been shown to be an excellent scaffold for producing high-performance oil absorbents with 3D porous structure, as it is composed of a microscale interconnected framework with good compressibility and is easy to chemically surface modify. Chen *et al*. directly carbonized melamine sponges at 1800 °C and successfully prepared superhydrophobic sponges with extraordinary absorbency^17^. Ruan *et al*. modified melamine sponges by grafting low surface energy tension fluorine-containing groups on the framework surface, the resultant sponge exhibited excellent performance for oil absorbency^18^. Yang *et al*. prepared a fire-resistant and compressible foam as a recyclable oil absorbent by pyrolizing melamine sponge at 400  °C and dipping in a hexane solution of chlorotrimethylsilane^19^. Pham *et al*. also fabricated superhydrophobic silanized melamine sponge by a toluene-immersion process^20^. Wang *et al*. protonated the melamine sponge for effective oil-water separation by soaking in a solution of 1.0 M HCl^21^. Along with chemical modification, incorporation of biomass, graphene and MoS_2_ have all been used to to modify melamine sponges from being hydrophilic to superhydrophobic and oleophilic. Yang *et al*. prepared a superoleophilic sponge as an oil absorbent by dip-coating the melamine sponge in a alkaline solution of lignin, followed by pyrolysis at 400  °C^22^. Nguyen *et al*. integrated solution-exfoliated graphene onto melamine sponges for oil absorbency, this was using a dip coating method^23^. This process made the sponge superhydrophobic and it showed excellent absorption capacities up to 165 times of the initial weight. Following this, Cho *et al*. altered the size distribution of graphene sheets using a grinding method in isopropyl alcohol and SDS, the fabricated graphene-coated melamine sponge delivered an absorption capacity of 153 g.g^−1^ for chloroform^15^. Graphene oxide (GO) was also introduced to modified melamine sponges followed by hydrothermal or chemical reduction to remove the oxygen-containing groups of GO^24^,^25^. The reduced GO-coated sponge exhibited adsorption capacity of 99 g.g^−1^ for diesel oil^24^. Another 2D material, MoS_2_, was used to decorate melamine sponges. Gao *et al*. exfoliated MoS_2_ nanosheets in ethanol and fabricated MoS_2_-coated melamine sponges by a dip-coating method in ethanol solution with the assistance of squeezing and vacuum degassing procedure^26^. Although, good absorption performance and relatively straightforward surface modifications procedures have been reported such as using dip coating methods, some drawbacks still remain in the preparation process of this technology and so limit its development, these include the generation of new pollutants, or high intrinsic material cost and using organics chemicals^10^,^12^,^13^,^27^. For graphene foams, harmful solvents, e.g. H_2_SO_4_ and HNO_3_, are used for the preparation of graphene oxide^27^. The uneconomic process of ultrahigh temperature, as high as several hundred, even 1000 °C are required for CNT growth or carbonization process of CNT sponges, pyrolized sponges^17^,^19^ and carbon aerogels^28^,^29^,^30^. Exfoliating graphene and MoS_2_ sheets requires organic solvent and also surfactant^15^,^23^,^26^. Developing environmentally benign, efficient and cost-effective new absorbent materials for the removal of oils from water remains desirable, yet challenging.

Herein, we report an organic-solvent-free, environmentally benign and cost-effective preparation method of a new absorbent material using a few-layer hBN-coated melamine sponge. A facile two-step strategy is applied to prepare the superhydrophobic sponge. First, hexagonal boron nitride (hBN), which is an isoelectric analog of graphene with high thermal conductivity, chemical stability and hydrophobicity^31^,^32^,^33^,^34^, is exfoliated to a few layers by probe sonication in water solution, thus obtaining a stable few-layer hBN dispersion. Subsequently, the commercially available melamine sponge (MS) was immersed in the hBN dispersion and coated with the aid of the interaction between the amine group in melamine (Lewis base) and the electron-deficient boron atoms on hBN (Lewis acid) at 90 °C^35^. The obtained hBN-coated melamine sponge (BMS) exhibits outstanding absorption performance, including high selectivity, excellent absorption capacities approaching 175 times its own weight and extraordinary recyclability reaching up to 80% absorption capacity after 30 load cycles. Moreover, this modified sponge could be used in conjunction with a vacuum apparatus for the continuous absorption and removal of oil pollutants from water surfaces, to enable high throughput oil pollutant removal. Due to its facile, environmental benign and cost-effective progress, we believe that such hBN-coated sponge with good performance will have great potential for real world practical applications.

## Results and Discussions

### Characterization of hBN dispersion

**Figure 1:**
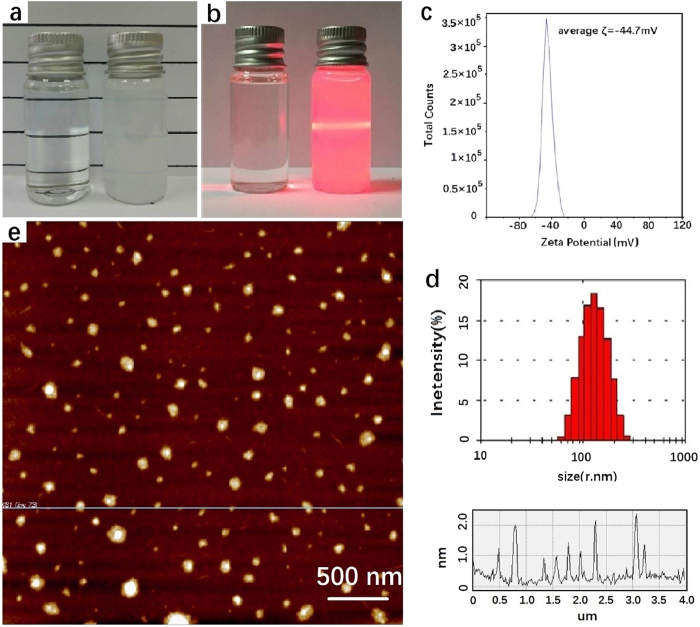
(**a**) Photographs of pure DI water (left) and h-BN nanosheets dispersed in DI water after two months of ageing. (**b**) The same solutions in (**a**) but with the irradiation of a red laser beam from the left. (**c**) zeta potential of h-BN dispersion. (**d**) Particle diameter histogram as measured by Dynamic Light Scattering. (**e**) AFM image (dimension: 4 × 4 μm) and the corresponding height profile of few-layered hBN.

The few-layered hBN dispersion was prepared using hBN powder as a starting source through probe sonication in deionized water for 8 hours and followed by centrifugation at 3000 rpm for 15 min to remove the residue. The as-obtained “milky” supernatant was similar in appearance to the reports on hBN dispersion in organic solvents^36^. The dispersion remained quite stable even after two months of ageing (Fig. 1a), which is consistent with the high zeta potential value of −44.7 mV (Fig. 1c). As shown in Fig. 1b, the path of a laser beam could be clearly seen through the hBN dispersion due to the scattering of the hBN nanosheets. The observed Tyndall effect in these solutions resemble what is found in graphene dispersions^37^ and functional hBN dispersions^38^. Dynamic light scattering (DLS) measurements and analysis showed a diameter ranging from 70–300 nm centered at around 130 nm in the aqueous hBN dispersion (Fig. 1d). Moreover, there was further evidence of the presence of few-layer hBN species, most with lateral sizes of 70–300 nm and having height lower than 2 nm, which was measured using noncontact (tapping) mode AFM, and is shown in Fig. 1e. In AFM studies of graphene, a height profile of around 1 nm is usually attributed to monolayer graphene due to the majority of the height value being the dead space between the substrate and atomic layer^39^,^40^. The measured thickness of the few-layer hBN of less than 2 nm, and indicates that the number of hBN layers is about 1–3 layers. Evidently, the hBN was successfully exfoliated by probe sonication to few-layer hBN.

### Characterization of hBN-coated sponge

**Figure 2:**
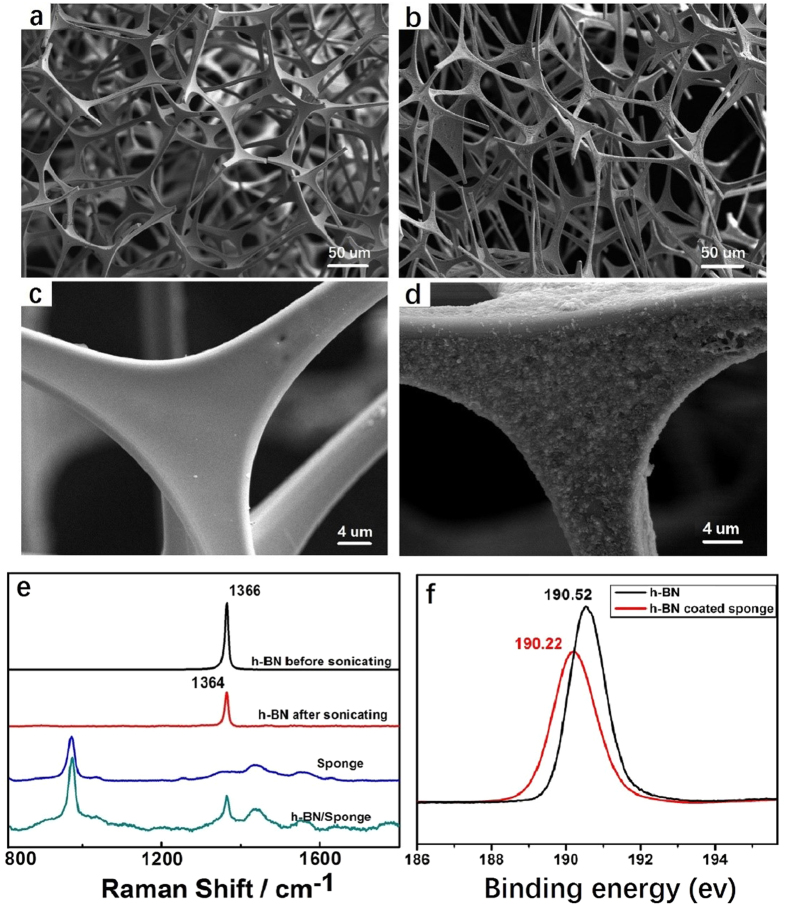
(**a**) Typical SEM images with different magnification of the pure virgin melamine sponges (**a** and **c**) and hBN-coated sponge(**b** and **d**). (**e**) Raman spectra of the bulk raw hBN power, exfoliated hBN (after sonication), pure sponge and hBN-coated sponge. (**f**) B1s XPS spectra of the exfoliated hBN and hBN-coated sponge.

The commercially available melamine sponge is an environmentally friendly material and is widely used in soundproofing and construction materials. The process for the preparation of hBN-coated melamine sponge is straightforward, only requiring direct immersion of a melamine sponge in a hBN dispersion at 90 °C for 24 hours. It was found that lower temperature and shorter immersion time led to an incomplete coverage of hBN on the melamine sponge and thus decreased the water contact angle, as shown in Figure S1. Higher temperature treatment, e.g. 120 °C made the sponge brittle, which is detrimental to its applicability in oil spills. Field emission scanning electron microscopy (FESEM) was successively used to examine the morphological evolution of the melamine sponge before and after coating with hBN nanosheets. As shown in Fig. 2a, the initially untreated melamine sponge has an interconnected network architecture with pore sizes ranging from 100–200 μm. The individual melamine fiber has a concave triangular shape with a smooth surface (Fig. 2c). After hBN deposition, the obtained sponge retains the three-dimensional (3D) interconnected network and a highly porous structure (Fig. 2b). None of the pores inside the melamine sponge are blocked. The open-pore network is beneficial for adsorption and maximizing the flow and uptake of oil and organic solvents. The magnified SEM image reveals that the smooth sponge skeletons were covered by hBN nanosheets, as shown in Fig. 2d. EDX result demonstrates that hBN exists on the surface of the melamine sponge (Figure S2). The successful deposition of hBN on the melamine sponge was also confirmed by Raman spectrometry (Fig. 2e). The pure melamine sponge and hBN nanosheets present Raman shift at 972 cm^−1^, 1450 cm^−1^ and 1365 cm^−1^, respectively. The hBN-coated melamine sponge shows all those characteristic peaks in the spectrum, which implies hBN existed on the melamine sponge. Notably, the Raman active E_2_ _g_ phonon mode for hBN powder and nanosheets are centered at 1367 cm^−1^ and 1365 cm^−1^, respectively. The red-shift of E_2_ _g_ phonon mode indicates the formation of few-layered hBN^34^, which is in accordance with the AFM results. The interaction between the melamine sponge and hBN was also evaluated by X-ray photoelectron spectroscopy (XPS). The observed binding energy of the B1s spectrum of exfoliated hBN and hBN-coated sponge is 190.52 eV and 190.22 eV (Fig. 2f), respectively, which evidently suggests the strong physical interaction between the melamine sponge and hBN. It is well documented that amine-containing molecules can be immobilized on the surface of hBN nanotubes and nanosheets due to the Lewis acid-base interaction between the electron lone-pair of nitrogen atoms in amine group and the vacant p-orbitals of the boron atoms in hBN^35^,^41^,^42^. The interaction between the electron-deficient boron atoms on the hBN and the electron-rich amine groups of melamine is responsible for the robust anchoring of hBN nanosheets on the surface of melamine sponge.

So far, there have been no reports detailing hBN-modified sponges. It is also worth noting that the entire fabrication procedure did not involve any organic solvents, expensive raw materials or sophisticated processing equipment. This indicates that our method is intrinsically cost-effective, environmentally benign and organic-solvent-free, and thus attractive in real world practical applications.

### Properties of hBN-coated sponge

**Figure 3:**
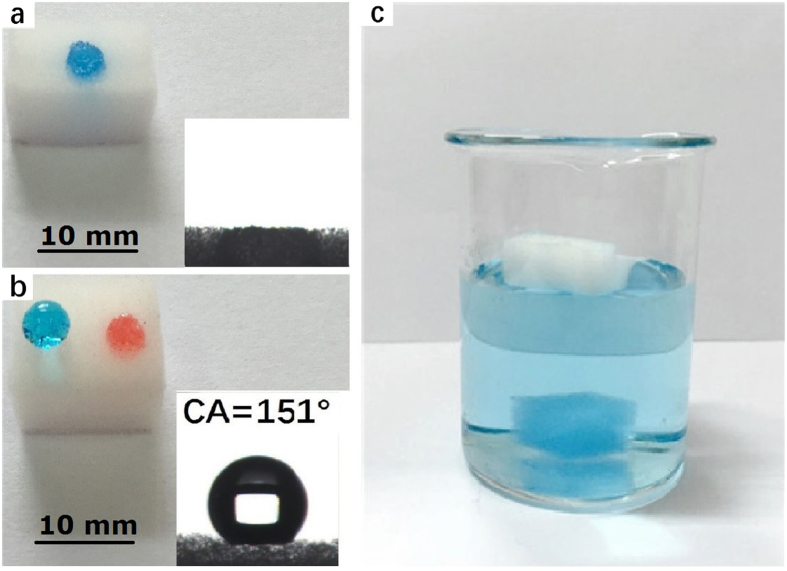
(**a**) The water droplet (colored with blue dye to facilitate observation) is completely absorbed by pure virgin melamine sponge and presents zero water contact angle (insert of a). (**b**) the water droplet is repelled from hBN-coated sponge and shows superhydrophobic character with a contact angle (CA) of 151°. The oil droplet (colored with orange dye) is completely absorbed by the hBN-coated sponge. (**c**) Photograph of the pure melamine sponge sinking to the bottom of the beaker and the corresponding hBN-coated sponge floating high on the water surface.

The surface wetting property of the melamine sponge before and after hBN deposition was examined by water contact angle (CA) measurements. The pure melamine sponge is superhydrophilic due to its abundant amine groups, as shown in Fig. 3a. A drop of water completely spread out and is immediately absorbed by the pure sponge. In contrast, the hBN-coated sponge displays a water contact angle of 151°, indicating superhydrophobic behavior (Fig. 3b). When pump oil was deposited on the surface of the hBN-coated sponge, it is immediately absorbed, as indicated by the circle marked in Fig. 3b, revealing its superoleophilic property. For comparison, when absorbents are placed on the surface of water, the pure melamine sponge quickly sinks to the bottom, but the hBN-coated sponge floats freely on the water surface, as exhibited in Fig. 3c. Generally, the wettability of the surface depends on the surface chemical composition and the surface topographical microstructure^43^,^44^. The synergy of the inherent hydrophobicity of hBN, the nanoscale roughness of the hBN deposits, and the porosity of sponge offers the superhydrophobicity and superoleophilicity of the hBN-modified melamine sponge. The results show that the hBN coating regulates the melamine sponge from its initial superhydrophilic state to being superhydrophobic and superoleophilic.

**Figure 4:**
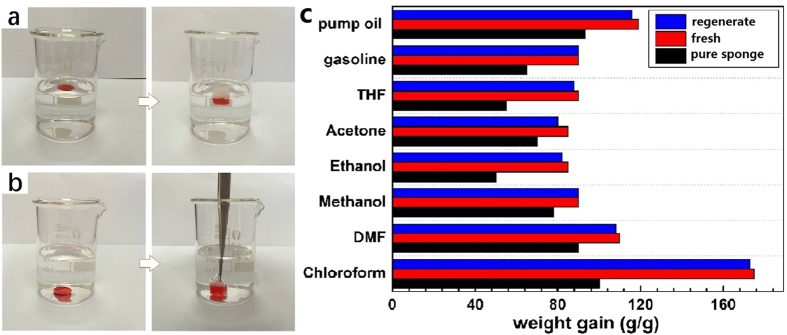
Digital photographs showing the adsorption of pump oil on the water surface (**a**) and chloroform in the bottom (**b**) by hBN-coated sponge. (**c**) The organic solvent adsorption capacity of pure sponge(black) fresh hBN-coated sponge (red) and the regenerated hBN-coated sponge (blue).

Its high porosity, strong hydrophobicity and superoleophilicity make the hBN-coated sponge a perfect candidate for the fast removal of various oils and organic solvents. As shown in Fig. 4a, a piece of hBN-coated sponge selectively and completely absorbed the pump oil (stained with Sudan red) from the water surface in a few seconds (Video S1), resulting in clean water originally contaminated by the oil. Similarly, when the hBN-coated sponge was immersed into the water to approach chloroform, the chloroform droplet was quickly removed by wicking (Fig. 4b, Video S2). The as-prepared hBN-coated sponge exhibits excellent absorption capacities towards a variety of oils and organic solvents, up to 90–175 times of its own weight depending on the density and viscosity of the liquid (Fig. 4c). The absorption capacity has greatly increased compared with pure sponge. Absorption of chloroform is almost two times as much as that of the pure sponge. Absorption capacity is obviously higher than many recent reported sorbents (Table 1), such as nitrogen-rich carbon aerogel^10^, CNT/PDMS coated PU sponge^27^, highly hydrophobic, fire-resistant (UHF) sponge^30^, cellulose nanofibril aerogels^45^ and micro-wrinkled reduced GO^46^. The capacity of hBN coated sponge is also higher than that of melamine sponge coated with exfoliated graphene (165 g.g^−1^, or 153 g.g^−1^)^15^,^23^, reduced graphene (99 g.g^−1^)^24^ and MoS_2_ (159 g.g^−1^)^26^. And the value is comparable to the state-of-the-art new sorbents with high absorption capacity, for example, twisted carbon fibers (TCF) aerogel^47^, carbon microbelts aerogel^48^, thiourea reduced graphene sponge^49^, CVD-produced CNT sponge^11^, CNT-graphene hybrid aerogel^50^. Although, the capacity is lower than that of few new materials, such as carbon sponge from melamine sponge^51^, cellulose nanofibril (CNF) aerogel^52^ and monolithic macroporous carbon (MMC) materials^53^, however, it should be noted that the fabrication process of the most of the reported materials need organic solvents, acids or high temperature, as demonstrated in Table 1. Importantly, the fabrication of hBN-coated sponge is perfectly green, environment-friendly and cost-effective, since there is no use of organic solvent, no contaminant disposal, no need for expensive raw material and sophisticated equipment. From the point of methodology, cost and efficiency, the present hBN-coated sponge has great potential application for the removal of chemical leakage.

**Figure 5:**
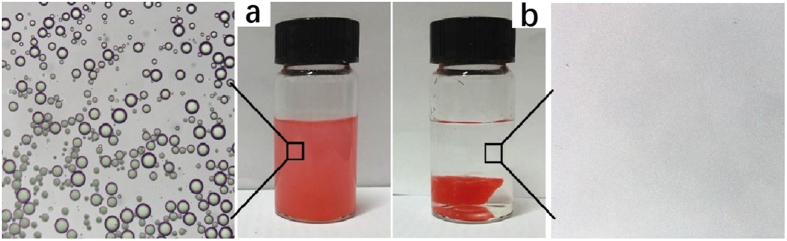
Photographs of toluene-in-water emulsion before and after hBN-coated sponge separation (toluene coloured with red dye).

In practical scenarios, such as chemical leakages, oils or organic solvents could emulsify in the water, thus complicating removal of the spilled products. The separation capability of the hBN-coated sponge was tested in such scenarios. An oil-in-water emulsion is obtained by sonicating a mixture of toluene and water. The optical microscope was used to record the images of the droplets in the original emulsions and after being treated by the hBN-coated sponge (Fig. 5). The results show the amount of emulsion present in the toluene-water mixture, this emulsion is absent after treatment with the hBN-coated sponge, which indicates the high efficiency in separating emulsified oil-water mixtures.

The as-prepared hBN-coated sponge also can be used for continuous oil-water separation. As illustrated in Figure S3, the hBN-coated sponge was used as a filter and connected to a vacuum pump system. When the sponge was placed at the water gasoline (labeled with red dye) interface, it was quickly filled with oil due to its selective oil absorption and water repellency caused by the hBN surface functionality conferring superhydrophobic and superoleophilic properties. Once the vacuum pump was turned on, gasoline was continuously absorbed by the hBN-coated sponge and was removed through the pipe and collected in the container (Video S3). Finally, the gasoline was removed completely, whilst water is completely rejected through the entire process, showing its strong selectivity. This experiment effectively demonstrates the excellent oil-water separation capability of the hBN-coated sponge.

**Figure 6:**
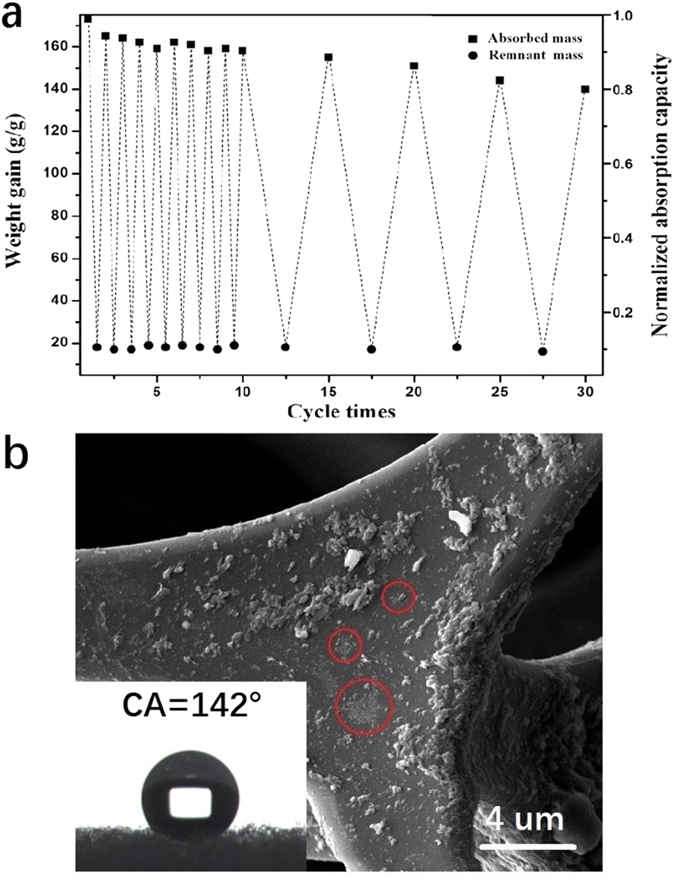
(**a**) Absorption capacity and residual amount of chloroform over 30 absorption/squeezing cycles. (**b**) Magnified SEM image of hBN-coated sponge after 30 cycles of absorption/squeezing tests. Inset in (**b**) is the corresponding surface water contact angle.

For practical applications, the recyclability of the absorbent and the recoverability of the absorbates are key criteria for the clean-up of oils or organic solvent spills because of economic and ecological demands for sustainability. Chloroform was used as the model absorbate to investigate the cyclic absorption/desorption behavior of the sponge. To verify the feasibility for practical applications, mechanical squeezing, the simplest desorption method, was chosen to release the absorbed liquid. In each cycle, nearly 90% of the absorbed chloroform was squeezed out mechanically. The absorption capability of the sponge remains 92% and has no apparent deterioration after 10 cycles of absorption/squeezing test. After 30 times absorption/squeezing cycles, the adsorption capability maintained over 80% (Fig. 6a). Importantly, the surface wetting property of the sponge was maintained, even after 30 cycles, as evidenced by the high water contact angle of 142° (inset of Fig. 6b). Figure 6b shows the magnified SEM image of the sponge after 30 cycles of absorption/squeezing. Some parts of hBN coating were destroyed by the repeated squeezing. However, most of the damaged area was still covered by the thin-layered hBN sheets (marked by the circle in Fig. 6b) due to the relatively robust anchoring of hBN nanosheets on the melamine sponge. The remaining hBN nanosheets and the nanoscale roughness contribute to the overall strong hydrophobicity of the sponge. Moreover, a repeatedly cycled sponge can be regenerated by simply washing the absorbate followed by immersion in a hBN dispersion at 90 °C for 24 h. After regeneration, the micromorphology is recovered as shown in Figure S4. The regenerated sponge exhibits nearly the same adsorption capacity, as shown in Fig. 4c. These results unambiguously demonstrate the excellent recyclability of the regenerated sponge.

## Conclusions

In summary, a superhydrophobic sponge with a few-layer hBN-coating was prepared through a simple sonication and immersion strategy. The hBN-coated sponge exhibited excellent absorption performance for oils and organic solvents, including good selectivity, high capacity and extraordinary recyclability. Based on these characteristics, the sponge was also demonstrated to be effective in the separation of oil-water emulsions and hydrodynamic oil-water separation. In particular, the fabrication of this absorbent is pollution-free and cost-effective, since it does not require any organic solvents, expensive raw materials, or sophisticated processing equipment. In the near future, such hBN-coated sponges could be scaled up and used in the field for the cleanup of oil spills and organic contaminant release.

## Method

hBN powder (99.9% metals basis, 1～2 μm) was purchased from Aladdin, melamine sponge was obtained from a local department store and used as received. Deionized water was prepared in the laboratory by ultra-pure water system (UPT-II-10T, Sichuan ULUPURE Ultra-pure Technology Co. Ltd.). All the chemicals were of analytical grade and used as received.

### Preparation of hBN dispersion and hBN-coated sponge

The pristine hBN powder (100 mg) was sonicated in deionized water (100 mL) at a concentration of 1 mg/mL in a round-bottomed beaker (150 mL) using a probe sonicator (JY92-IIDN, NingBo Scientz Biotechnology co., LTD) for 8 h. The resultant white slurry was centrifuged at 3000 rpm for 15 min in order to remove residual large-sized hBN particles. The supernatant of exfoliated h-BN nanosheets was collected and allowed to equilibrate for 24 h to allow any insoluble material or aggregates to precipitate. Finally, the milky white supernatant fraction was retained.

The commercial melamine sponge was cut into blocks, and ultrasonically cleaned in ethanol and deionized water, respectively. Then the blocks were washed several times with deionized water. Finally, the pretreated melamine sponge was dried in air at 60 °C for 24 h in an oven. The prepared sponges were immersed into the few-layered hBN dispersion for 24 h at 90 °C, then dried in the vacuum oven for 12 h to ensure completely removal of DI water by evaporation. Finally, exfoliated hBN nanosheets were physically coated on the melamine sponge.

### Characterizations

The optical images were captured by a digital camera (Canon). Malvern Zetasizer NanoZS90 was used to measure the zeta potential of the hBN nanosheets in the dispersion. Dynamic light scattering (DLS) is the most widely used to measure nanoparticle size. Atomic force microscopy (NT-MDT) was employed in noncontact (tapping mode) mode to investigate the surface morphologies of the hBN films. The porous structures of melamine sponges and hBN-coated sponges were observed using Scanning electron microscopy (MERLIN Compact, Carl Zeiss Jena). Before observation, the samples were coated with gold using a sputtering coater. Raman spectra (Invia Renishaw) were measured for the hBN powder, pure melamine sponge and hBN/sponge. X-ray photoelectron spectroscopy (XPS) was performed on the Thermo Scientific ESCALab 250Xi using 200 W monochromated Al Kα radiation. The 500 μm X-ray spot was used for XPS analysis. The base pressure in the analysis chamber was about 3 × 10^−10^ mbar. Typically the hydrocarbon C1s line at 284.8 eV from adventitious carbon is used for energy referencing. The contact angle (CA) was measured with 5 μL droplets of water using a contact angle apparatus (POWEREACH-JC2000C1) at ambient temperature. The absorption capacities of hBN/sponges for various organic solvents were measured. A weighed amount of hBN sponge was put into a beaker containing the organic solvent and allowed to saturate by absorption. Then absorption capacities could be calculated through the definition: (W_saturated absorption_-W_initial_)/W_initial_. In the cyclic absorption/squeezing measurement, the hBN coated sponge with volume about 10 × 10 × 10 mm^3^ was dropped into the organic solvent until the absorbent was completely filled with the liquid, and then it was taken out and quickly weighed to avoid evaporation of the absorbate (W_saturated absorption_). After that, a counterweight of 2000 g was put on the top of the sponge to squeeze out the liquid and the sponge was compressed by about 90%. The total weight after squeezing out the absorbate (W_squeeze_, including the weight of the absorbent and the residual absorbate) was measured again to calculate the remnant capacity by the definition: (W_saturated absorption_-W_squeeze_)/W_squeeze_.

## Additional Information

**How to cite this article**: Zhou, Y. *et al*. Superhydrophobic hBN-Regulated Sponges with Excellent Absorbency Fabricated Using a Green and Facile Method. *Sci. Rep.* **7**, 45065; doi: 10.1038/srep45065 (2017).

**Publisher's note:** Springer Nature remains neutral with regard to jurisdictional claims in published maps and institutional affiliations.

## Supplementary Material

Supporting Information

Supplementary Video S1

Supplementary Video S2

Supplementary Video S3

## Figures and Tables

**Table 1:** Comparison of various sorbent materials.

Sorbent materials	Absorbed substances	Absorption capacity (g/g)	cost	method	Circles and remained rate %	ref.
Nitrogen-rich carbon aerogel	oils and organic solvents	5–16	low	High temperature pyrolysis (700 °C), organic solvent	100, 61.2%	10
CNT/PDMS coated PU sponge	oils	15–25	low	Organic solvent	n.a.	27
cellulose nanofibril aerogel	oils and organic solvents	25–50	high	Complicated, organic solvent	5, 60%	45
Graphene sponge (glucose)	oils and organic solvents	23–35	low	Polymer, H_2_SO_4_, HCl	100, 75%	54
micro-wrinkled reduced GO	oils	40–80	low	Complicated, acid and alkali		46
Graphene melamine Sponge	diesel oil	99	high	Complicated, H_2_SO_4_, hydrazine hydrate	n.a.	24
Ultralight fire-resistant sponge	oils and organic solvents	55–145	Low	Organic solvent, nickel	5, 70%	30
Few Layer Graphene-sponges	oils and organic solvents	57–153	high	Complicated,	20, 99%	15
P2VP-b-PHA Graphene sponge	oils and organic solvents	50–200	high	Organic solvent, polymer	10, 97%	13
twisted carbon fibers aerogel	oils and organic solvents	50–192	low	High temperature pyrolysis (800 °C)	5, 80%	47
Carbon microbelts aerogel	oils and organic solvents	56–188	low	High temperature pyrolysis (850 °C)	n.a	48
Graphene sponge (thiourea as the reducing reagent)	oils and organic solvents	60–160	high	Hydrothermal, Complicated	5, 93%	49
CNT sponge(CVD)	oils and organic solvents	80–180	low	High temperature (860 °C) CVD, organic solvent	n.a.	11
CNT-graphene hybrid aerogel	oils	90–140	high	Complicated	5, 75%	50
MoS_2_ Sponge	oils and organic solvents	82–159	low	Dip in the ethanol	20, 90%	26
Carbon sponge from MS	oils and organic solvents	90–200	high	High temperature pyrolysis (300–800 °C)	n.a.	51
Monolithic macroporous carbon materials	oils and organic solvents	87–273	low	High temperature (500 °C)	5, 98%	53
cellulose nanofibril aerogel	oils and organic solvents	106–312	low	High temperature (700–1300 °C)	5, 89%	52
Lignin-adsorbed sponge	oils and organic solvents	90–217	low	Alkaline solution, pyrolyzed (400 °C)	5, 99%	22
hBN-coated sponge	oils and organic solvents	90–175	low	Water sonication	30, 80%	present work
