# Supplementary material for: Superhydrophobic hBN-Regulated Sponges with Excellent Absorbency Fabricated Using a Green and Facile Method
Source: Sci Rep. 2017 Mar 23;7:45065. doi: 10.1038/srep45065 (PMC5362905; doi:10.1038/srep45065)
Supplement: Supporting Information [file srep45065-s1.pdf]

Supporting Information

Superhydrophobic hBN-Regulated Sponges with Excellent Absorbency Fabricated Using a Green and Facile Method

Ying Zhou[a], Yao Wang[a], Tengfei Liu[a], Gang Xu[b], Guangming Chen[c], Huayi Li[c], Lichun Liu[d], Qiqi Zhuo[a], Jiaoxia Zhang[a], Chao Yan[a]*

a. *School of Materials Science and Engineering, Jiangsu University of Science and Technology, Zhenjiang, 212003, P R China.*
b. *School of Naval Architecture and Ocean Engineering, Jiangsu University of Science and Technology, Zhenjiang, 212003, P R China.*
c. *Institute of Chemistry, Chinese Academy of Science, Beijing, 100190, P R China*
d. *College of Biological, Chemical Sciences and Engineering, Jiaxing University, Jiaxing, 314001, P R China*

* *Corresponding author: Chao Yan, Email: chaoyan@just.edu.cn*

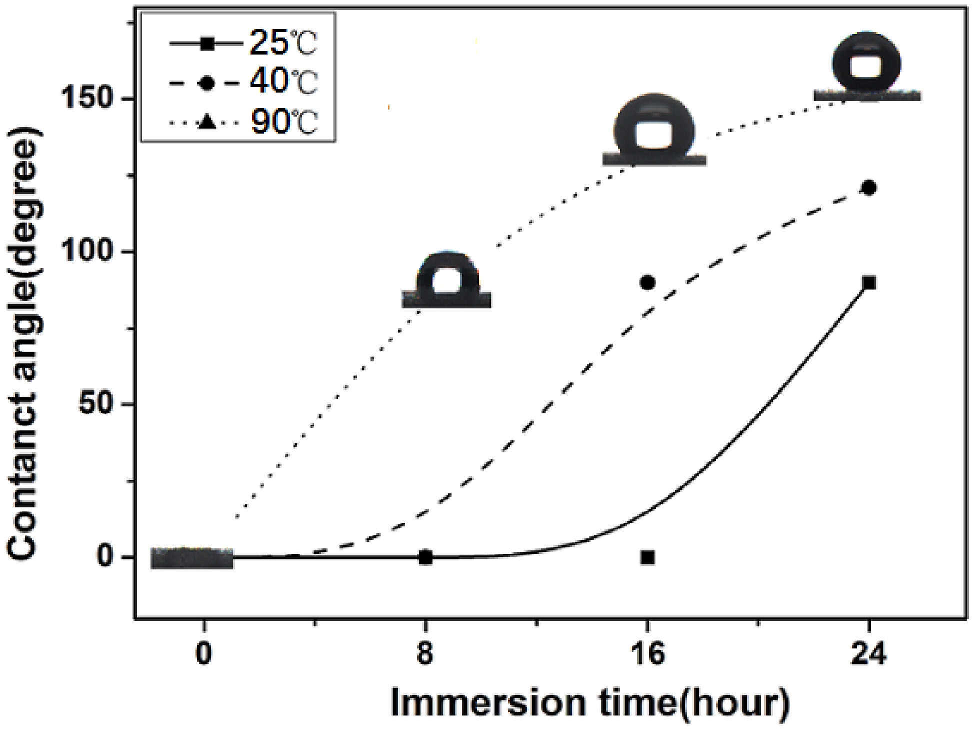

Figure S1 The contanct angle on h-BN coated h-BN at different immersion time(0h, 8h, 16h, 24h) and temperature(25^0C, 40^0C, 90^0C). The contanct angle of h-BN coated sponge increased with the longer time and higher temperature.

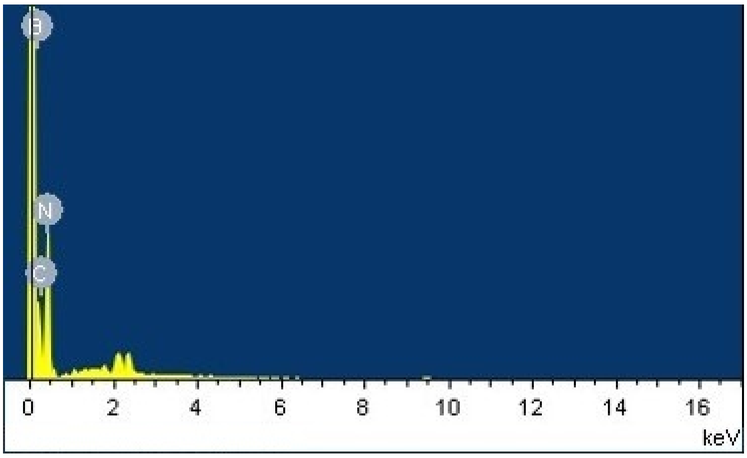

Figure S2. EDX of hBN-coated sponge.

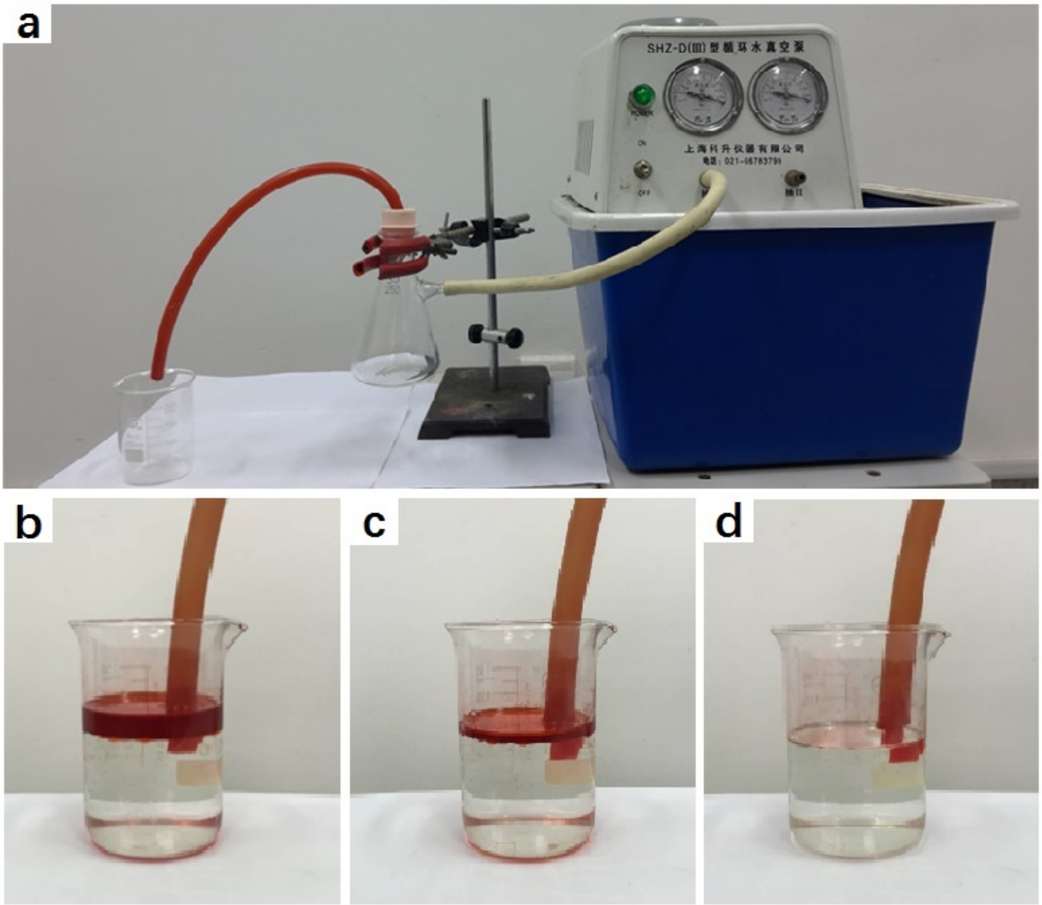

Figure S3. Digital photographs of (a) the continuous oll-water separation system, (b-d) the progress of continuous revomal of gasoline from oil-water system.

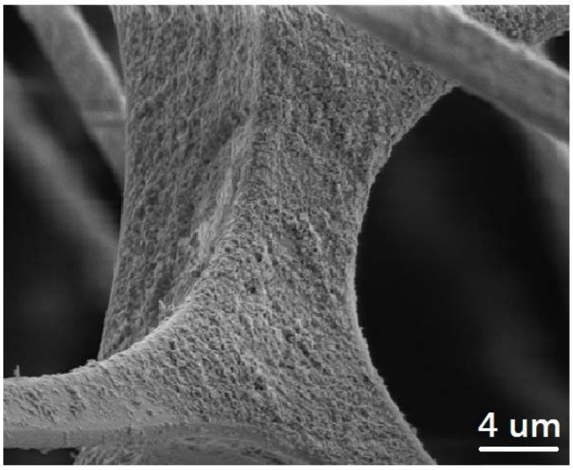

Figure S4. Magnified SEM image of regenerated hBN-coated sponge.

Video S1. The absorption process of hBN-decorated sponge for pump oil on water surface.

Video S2. The absorption process of hBN-decorated sponge for chloroform under

water.

Video S3. The process of hBN-decorated sponge for continuous oil/water separation.
